# Minimal residual disease status improved the response evaluation in patients with Waldenström’s macroglobulinemia

**DOI:** 10.3389/fimmu.2023.1171539

**Published:** 2023-05-10

**Authors:** Wenjie Xiong, Zanzan Wang, Tingyu Wang, Ying Yu, Yanshan Huang, Hao Sun, Jiawen Chen, Rui Lyu, Huijun Wang, Yuting Yan, Qi Wang, Wei Liu, Gang An, Weiwei Sui, Wenyang Huang, Dehui Zou, Zhijian Xiao, Jianxiang Wang, Guifang Ouyang, Lugui Qiu, Shuhua Yi

**Affiliations:** ^1^ State Key Laboratory of Experimental Hematology, National Clinical Research Center for Blood Diseases, Institute of Hematology & Blood Diseases Hospital, Chinese Academy of Medical Sciences & Peking Union Medical College, Tianjin, China; ^2^ Department of Lymphma & Myeloma, Haihe Laboratory of Cell Ecosystem, National Clinical Research Center for Blood Diseases, Institute of Hematology & Blood Diseases Hospital, Chinese Academy of Medical Sciences & Peking Union Medical College, Tianjin, China; ^3^ Tianjin Institutes of Health Science, Tianjin, China; ^4^ Department of Hematology, Ningbo First Hospital, Ningbo, China

**Keywords:** Waldenström’s macroglobulinemia, minimal residual disease, multiparameter flow cytometry, prognosis, remission

## Abstract

**Introduction:**

Minimal residual disease (MRD) has been recognized as an important prognostic factor of survival in patients with hematological malignancies. However, the prognostic value of MRD in Waldenström macroglobulinemia (WM) remains unexplored.

**Methods:**

We analyzed 108 newly diagnosed WM patients receiving systematic therapy and assessed for MRD by multiparameter flow cytometry (MFC) using bone marrow samples.

**Results:**

Of the total patients, 34 (31.5%) achieved undetectable MRD (uMRD). A hemoglobin level of >115 g/L (P=0.03), a serum albumin level of >35 g/L (P=0.01), a β2-MG level of ≤3 mg/L (P=0.03), and a low-risk International Prognostic Scoring System for WM (IPSSWM) stage (P<0.01) were associated with a higher rate of uMRD. Improvements in monoclonal immunoglobulin (P<0.01) and hemoglobin (P=0.03) levels were more evident in uMRD patients compared with that in MRD-positive patients. The 3-year progression-free survival (PFS) was better in uMRD patients compared with that in MRD-positive patients (96.2% vs. 52.8%; P=0.0012). Landmark analysis also showed that uMRD patients had better PFS compared with MRD-positive patients after 6 and 12 months. Patients who achieved partial response (PR) and uMRD had a 3-year PFS of 100%, which was significantly higher than that of patients with MRD-positive PR (62.6%, P=0.029). Multivariate analysis showed that MRD positivity was an independent factor of PFS (HR: 2.55, P=0.03). Moreover, the combination of the 6th International Workshop on WM assessment (IWWM-6 Criteria) and MRD assessment had a higher 3-year AUC compared with the IWWM-6 criteria alone (0.71 vs. 0.67).

**Discussion:**

MRD status assessed by MFC is an independent prognostic factor for PFS in patients with WM, and its determination could improve the precision of response evaluation, especially in patients who achieved PR.

## 1 Introduction

Waldenström’s macroglobulinemia (WM) is defined as lymphoplasmacytic lymphoma with bone marrow (BM) involvement and presence of monoclonal immunoglobulin (IgM) at any concentration (1). The treatment of WM, including monoclonal antibodies with chemotherapy or Bruton’s tyrosine kinase (BTK) inhibitors, can achieve high response rates and prolong the progression-free survival (PFS) (2–4). Given the availability of efficient therapies, an enhanced assessment of treatment response is highly desirable.

The evaluation of total serum IgM levels plays an important role in the assessment of responses according to the 6th International Workshop on WM (5). However, the categorical response definitions have some limitations. It is difficult to assess the response of WM patients with low levels of IgM, especially those who achieve near complete response (CR). In the current response assessment system, BM assessment is not routinely recommended, except in cases where confirmation of CR is needed. The prognostic value of BM remission throughout the course of treatment remains unclear. Furthermore, the BM response and IgM levels vary based on the treatment used. Several studies have demonstrated fluctuations and delays in IgM and rapid BM responses in patients receiving a rituximab-based regimen (6, 7). Conversely, modest BM burden changes and a deep improvement in IgM levels occur in patients receiving proteasome inhibitor-based regimens (8). Therefore, it is necessary to develop sensitive technologies that can detect BM infiltration to assess for treatment response.

Minimal residual disease (MRD) status has been increasingly recognized as an important prognostic factor in several hematological malignancies, such as chronic lymphocytic leukemia, multiple myeloma, and acute promyelocytic leukemia (9–12). However, only a few studies have systematically investigated the impact of MRD detected using multiparameter flow cytometry (MFC) on the response assessment and outcome in WM patients. In the present study, we aimed to explore whether MRD is a sensitive indicator for evaluating both response and outcome.

## 2 Materials and methods

### 2.1 Patients

This study was approved by the Ethics Committee of the Institute of Hematology and Blood Disease Hospital and complied with the Declaration of Helsinki. The diagnosis was confirmed according to the diagnostic criteria from the Second International Workshop on WM (13). Finally, 108 patients who underwent a full course of treatment and achieved an MRD status were included in this prospective study from January 2010 to November 2022. All patients were newly diagnosed and had at least one symptomatic disease that met the recommendations for treatment according to the 2nd International Workshop on WM. Patients who had an Eastern Cooperative Oncology Group performance status (ECOG-PS) score of 0–2 and a life expectancy of >3 months were also included. Patients with serious complications, liver or renal lesions unrelated to lymphoma, clinical nervous system dysfunction, human immunodeficiency virus infection, active hepatitis B virus infection, or other uncontrolled systemic infections were excluded. The flow chart of the patient selection process is shown in **Figure S1**. Written informed consent was obtained from all participants.

### 2.2 Data collection

Data on patients’ age, sex, ECOG PS score, B symptoms, hemoglobin levels, platelet counts, serum β2-microglobulin level, serum IgM level, protein electrophoresis status, lactate dehydrogenase (LDH) level, albumin level at therapy initiation, and best response were retrospectively retrieved. Extramedullary disease was defined as lymphadenopathy of ≥1.5 cm in diameter on computed tomography (CT) scan or splenomegaly of ≥13 cm in the largest axis on ultrasound. The results of flow cytometer detection and analysis of histological sections obtained from each patient were reviewed by at least two hematologists and pathologists.

### 2.3 Flow cytometry sample preparation and MRD assessments

Bone marrow samples were collected from the patients and placed in a container with dipotassium ethylene diamine tetraacetic acid anticoagulant; the mature red blood cells were lysed within 24 h using 2 ml of 1× lysing buffer (BD Biosciences, San Jose, CA, USA) without fixative. Fresh cells (1×10^7^ cells) were isolated from the bone marrow samples through Ficoll separation and analyzed prospectively using eight-color flow cytometry. The samples from healthy individuals were used as negative controls. Sample preparation and flow cytometer detection were conducted following the manufacturer’s recommendations and as previously described (9, 14). MRD was determined by eight-color flow cytometry in bone marrow samples after two and/or four courses following the induction of therapy, at the end of treatment, and during the assessment of best response. For patients receiving continuous BTK inhibitors treatment, the end of induction therapy was performed at the end of 6 courses which was as the same as immunochemotherapy. In order to exclude the interference of treatment, the MRD at best response was selected for final survival analysis. And the MRD detected at best response is referring to bone marrow (MRD) testing performed at the time of best IgM response. An MRD positive result was defined as a cluster of >50 clonal malignant cells identified in 500,000 nucleated cells. An MRD negative result was defined as a clonal malignant cell count of <10^−4^ (0.01%). Two tubes of an eight-color panel containing the following antibody markers were used: Lambda-FITC/CD10-PE/CD5-PerCP-Cy5.5/CD38-PE-Cy7/Kappa-APC/CD20-APC-H7/CD19-V450/CD45-V500 and cLambda-FITC/CD138-PE/CD56-PerCP-Cy5.5/CD38-PE-Cy7/cKappa-APC/CD117-APC-H7/CD19-V450/CD45-V500. The antibodies were purchased from Becton-Dickinson (San Jose, CA, USA), Beckman-Coulter (Brea, CA, USA), DAKO (Troy, MI, USA), and BioLegend (San Diego, CA, USA). The MFC was measured on a FACSCanto II flow cytometer (Becton Dickinson, BD) using the Kaluza™ software (Beckman-Coulter).

### 2.4 Assessment of treatment response

The treatment response criteria were established according to the National Comprehensive Cancer Network guidelines (15), which were modified from the 6th International Workshop on WM (IWWM-6 Criteria) (5).

### 2.5 Statistical analysis

Disease progression within 24 months (POD24) was defined as the first progression of disease that occurred within 24 months after the initiation of first-line therapy. PFS was defined as period from the initiation of treatment to the disease progression, last follow-up, or death from any cause. Overall survival (OS) was defined as the period from the initiation of treatment to the last follow-up or death from any cause. The PFS and OS were calculated using the Kaplan-Meier method and compared using the log-rank test. Landmark analyses of PFS in different MRD groups were performed according to the median and maximum time until they achieved undetected MRD (uMRD). The median time to achievement of uMRD was 6 months (range, 2–12 months). Therefore, the cutoff points for the landmark analysis were set at 6 and 12 months. The landmark analyses of PFS in the uMRD partial response (PR) and MRD-positive PR subgroups were based on the results of the analyses of MRD, and the cutoff points were also set at 6 and 12 months. For each type of event, the patients were excluded from the analysis at the time of the first event; for example, a patient who experienced an incident that resulted in disease progression or death within the first 6 or 12 months was excluded from the analysis at the time of the event and in the subsequent months after the landmark point. Landmark analyses were performed overall and after stratification of events according to their association with the events. The chi-square test or Fisher’s exact test was used to assess differences between categorical variables. Continuous variables were compared using the Mann-Whitney U test. Concordance was assessed using the overall percentage agreement and Cohen’s κ coefficient. Multivariate analysis was performed using Cox regression models. Statistical analyses were performed using the Statistical Package for the Social Sciences version 25.0 (IBM Corporation, Armonk, NY), GraphPad Prism software version 7.0 (La Jolla, CA), and R statistical program version 4.0.5.

## 3 Results

### 3.1 Patients’ characteristics

The baseline characteristics of the 108 patients are described in **Table 1**. The median age was 59 years (range, 33–80 years), while 23 patients (21.3%) were aged >65 years. Four patients (3.7%) were aged >75 years. The male/female ratio was 1.9 (71/37). Moreover, 97 patients (89.8%) had anemia (≤11.5 g/dL), while 35 patients (32.4%) had thrombocytopenia (≤100×10^9^/L). The median level of IgM was 34 g/L (range, 6.1–122.0 g/L). Using the IPSSWM staging system, 15 patients (13.9%) were categorized as the low-risk group, 40 (37.0%) as the intermediate-risk group, and 35 (32.4%) as the high-risk group. MYD88^L265P^ mutation was detected in 85 patients (78.7%), and no significant difference was observed between the uMRD and MRD-positive groups.

**Table 1:** The comparison of clinical characteristics between uMRD and MRD-positive patients.

Characteristic	Overall	uMRD	MRD-positive	*P*
N	108	34	74	
Age (years)				
Median (range)	59 (33-80)	58 (33-80)	61 (33-77)	
<65	85 (78.7%)	30 (88.2%)	55 (74.3%)	0.13
≥65	23 (21.3%)	4 (11.8%)	19 (25.7%)	
Gender				
Female	37 (34.3%)	14 (41.2%)	23 (31.1%)	0.38
Male	71 (65.7%)	20 (58.8%)	51 (68.9%)	
ECOG				
0-1	94 (87.0%)	30 (93.8%)	64 (91.4%)	0.99
≥2	8 (7.4%)	2 (6.2%)	6 (8.6%)	
B symptoms				
Absent	72 (66.7%)	23 (74.2%)	49 (72.1%)	0.99
Present	27 (25.0%)	8 (25.8%)	19 (27.9%)	
Hb (g/dL)				
Median (range)	84 (25-143)	92 (34-143)	81 (25-134)	
>11.5	9 (8.3%)	6 (17.6%)	3 (4.2%)	**0.03**
≤11.5	97 (89.8%)	28 (82.4%)	69 (95.8%)	
PLT (10^9^/L)				
Median (range)	141 (4-481)	163 (16-481)	135 (4-405)	
>100	69 (63.9%)	23 (67.6%)	46 (65.7%)	0.99
≤100	35 (32.4%)	11 (32.4%)	24 (34.3%)	
β2-MG (mg/L)				
Median (range)	3.8 (1.9-12.6)	3.4 (1.9-8.8)	4.1 (1.9-12.6)	
≤3	27 (25.0%)	14 (45.2%)	13 (21.0%)	**0.03**
>3	66 (61.1%)	17 (54.8%)	49 (79.0%)	
LDH (U/L)				
Median (range)	154 (69-874)	184 (73-874)	146 (69-523)	
<250	76 (70.4%)	24 (75.0%)	52 (88.1%)	0.14
≥250	15 (13.9%)	8 (25.0%)	7 (11.9%)	
IgM (g/L)				
Median (range)	34 (6.1-122.0)	25 (6.1-122.0)	37 (6.7-119.0)	
≤70	85 (78.7%)	30 (90.9%)	55 (79.7%)	0.26
>70	17 (15.7%)	3 (9.1%)	14 (20.3%)	
IPSSWM				
Low-risk	15 (13.9%)	10 (32.2%)	5 (8.5%)	**<0.01**
Median-risk	40 (37.0%)	15 (48.4%)	25 (42.4%)	
High-risk	35 (32.4%)	6 (19.4%)	29 (49.2%)	
Serum Albumin (g/L)				
Median (range)	34 (21-47)	37 (25-44)	33 (21-47)	
≥35	48 (44.4%)	22 (64.7%)	26 (37.7%)	**<0.01**
<35	55 (50.9%)	12 (35.3%)	43 (62.3%)	
Lymphadenopathy (cm)				
<1.5	69 (63.9%)	24 (70.6%)	45 (60.8%)	0.39
≥1.5	39 (36.1%)	10 (29.4%)	29 (39.2%)	
Splenomegaly(cm)				
<13	86 (79.6%)	29 (85.3%)	57 (77.0%)	0.44
≥13	22 (20.4%)	5 (14.7%)	17 (23.0%)	
Regimen				
R-based therapy	53 (49.1%)	24 (70.6%)	29 (39.2%)	**<0.01**
PI-based therapy	20 (18.5%)	7 (20.6%)	13 (17.6%)	
BTK-inhibitor	19 (17.6%)	1 (2.9%)	18 (24.3%)	
Traditional chemotherapy	4 (3.7%)	0 (0%)	4 (5.4%)	
R+PI based therapy	12 (11.1%)	2 (5.9%)	10 (13.5%)	
Maintenance therapy				
No	50 (46.3%)	13 (38.2%)	37 (50%)	0.30
Yes	58 (53.7%)	21 (61.8%)	37 (50%)	
POD24				
Negative	92 (85.2%)	34 (100%)	58 (78.4%)	**<0.01**
Positive	16 (14.8%)	0 (0%)	16 (21.6%)	
Best response				
CR	8 (7.4%)	8 (23.5%)	0 (0%)	**<0.01**
VGPR	16 (14.8%)	8 (23.5%)	8 (10.8%)	
PR	56 (51.8%)	18 (53.0%)	38 (51.4%)	
MR	14 (13.0%)	0 (0%)	14 (18.9%)	
SD	14 (13.0%)	0 (0%)	14 (18.9%)	
MYD88 mutation	85 (78.7%)	26 (76.5%)	59 (79.7%)	0.47

uMRD, undetectable minimal residual disease; IgM, Monoclonal immunoglobulin M; ECOG, Eastern Cooperative Oncology Group; Hb, hemoglobin; PLT, platelet; β2-MG, β2-microglobulin; LDH, lactic dehydrogenase; IPSSWM, International Scoring System for Waldenström’s Macroglobulinemia; MRD, minimal residual disease; R, rituximab; PI, proteasome inhibitor; POD24, time to progression of disease within 24 months; CR, complete response; VGPR, very good partial response; PR, partial response; MR, minor response; SD, stable disease. Significant P-values are in bold font.

### 3.2 Treatment

Of the 108 patients included, 53 (49.1%) received rituximab-based induction therapies, while 20 (18.5%) received proteasome inhibitor-based regimens. Furthermore, 19 patients (17.6%) received BTK inhibitors. Twelve patients (11.1%) received proteasome inhibitor-rituximab combination therapy, while only four patients (3.7%) received traditional chemotherapies. The median number of treatment courses was 6 (range, 3–10 courses). After the completion of induction therapy, 58 patients (53.7%) received maintenance therapy with rituximab or thalidomide, but only 50 patients (46.3%) were observed.

### 3.3 Response to treatment

The best overall response rate (ORR) was 87.0%, with eight patients (7.4%) achieving a CR, 16 patients (14.8%) achieving a very good partial response (VGPR), 56 patients (51.8%) showing a partial response (PR), and 14 patients (13.0%) showing a minor response (MR). Fourteen patients (13.0%) had stable disease (SD). The median time to respond was 1.8 months (range, 1–9 months). The median time to best response was 6 months (range, 2–12 months). CT-detected lymphadenopathy (≥1.5 cm) was reported in 39 of 108 patients at baseline. In addition, 18 patients (46.2%) demonstrated complete resolution of lymphadenopathy, while 12 patients (30.8%) only demonstrated partial resolution of lymphadenopathy. Among 22 patients with splenomegaly, 15 (68.2%) showed complete resolution, while seven patients (31.8%) only showed partially resolution after chemotherapy.

### 3.4 MRD response

Before treatment, 99 patients (91.7%) had a tumor burden by MFC of >1.0%, and 9 patients (8.3%) had tumor burden by MFC of 0.01%–1%. After two courses of induction therapy, 89 patients (82.4%) had available MRD results. Seven patients (6.5%) had uMRD, 31 patients (28.7%) attained an MRD rate of 0.01%–1%, and 51 patients (47.2%) attained an MRD rate of >1.0%. After four courses of induction therapy, 99 patients (91.7%) had available MRD results. In addition, 21 patients (19.4%) attained uMRD, 29 patients (26.9%) attained an MRD rate of 0.01%–1%, and 49 patients (45.4%) attained an MRD rate of >1.0%. After induction therapy, the MRD results were available in 100 patients (92.6%). Moreover, 31 patients (28.7%) had uMRD, 30 patients (27.8%) attained an MRD rate of 0.01%–1%, and 39 patients (36.1%) attained an MRD rate of >1.0%. At best response, 34 patients (31.5%) had uMRD, 34 patients (31.5%) attained an MRD rate of 0.01%–0.1%, and 40 patients (37.0%) attained an MRD rate >1% (**Figure S2**).

### 3.5 Factors associated with uMRD status

Pretreatment characteristics were evaluated to determine the factors associated with uMRD status when the best response was achieved. A hemoglobin level of >11.5 g/dL (17.6% vs. 4.2%, *P*=0.03), a serum albumin level of ≥35 g/L (64.7% vs. 37.7%, *P*<0.01), a β2-MG level of ≤3 mg/L (45.2% vs. 21.0%, *P*=0.03), and a low-risk IPSSWM stage (32.2% vs. 8.5%, *P*<0.01) were significantly associated with a higher rate of uMRD. Rituximab-based chemotherapies showed a preferential MRD clearance rate compared with proteasome inhibitor-based regimens (70.6% vs. 20.6%, *P*<0.01). Additionally, 16 of 74 patients (21.6%) in the MRD-positive group had POD24, while none of the 34 patients with uMRD experienced POD24 (*P*<0.01). However, no difference was found in the distribution of age, sex, ECOG PS score, B symptoms, platelet count, LDH level, or IgM level between the uMRD and MRD-positive groups (**Table 1**).

### 3.6 MRD reflecting the changes in tumor burden

**Figure 1:**
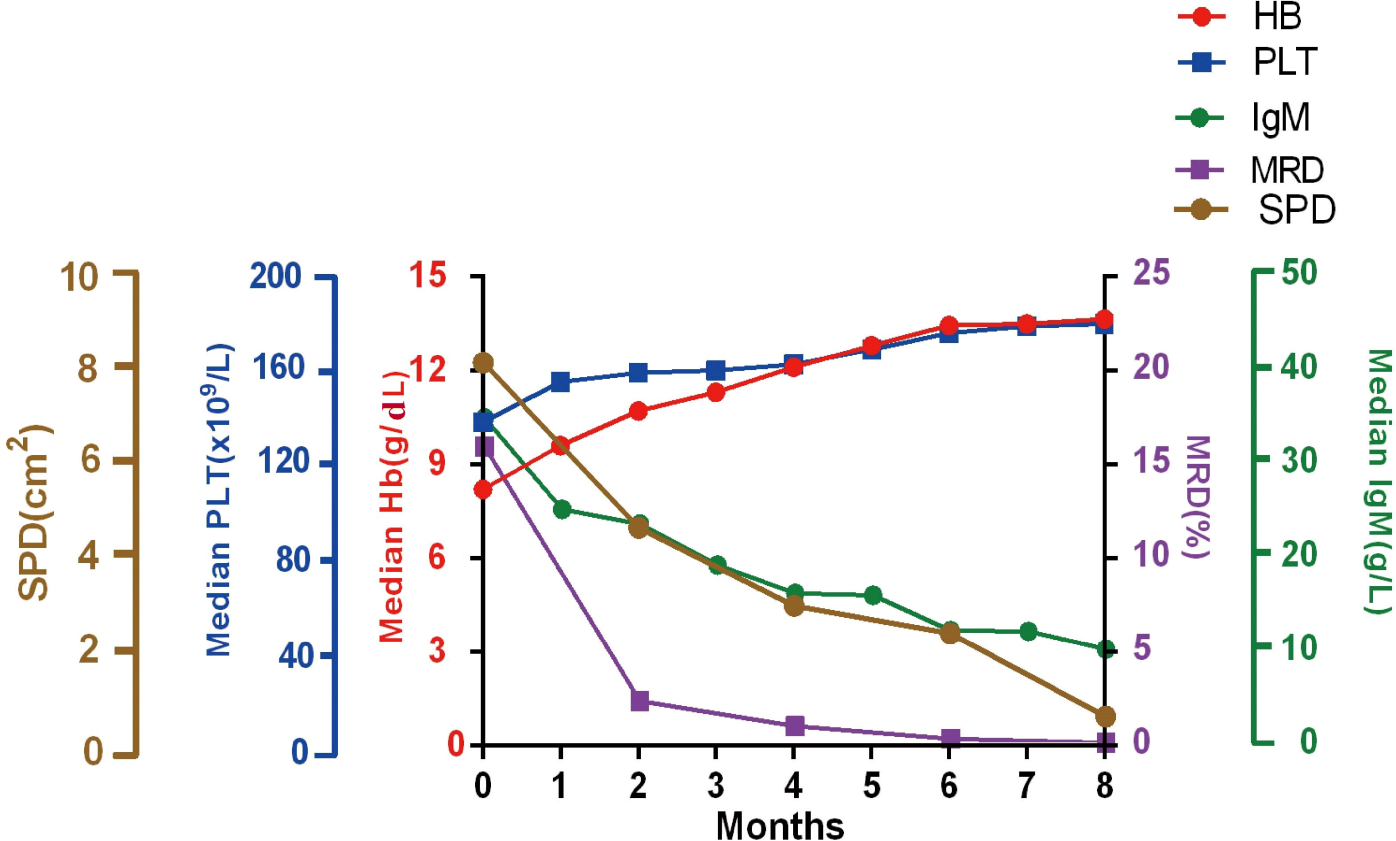
Improvement in clinical characters. Median hemoglobin, platelet counts, serum IgM, the SPD of lymphadenopathy, and MRD responses during induction treatment. Hb, hemoglobin; PLT, Platelets; SPD, the sum of the product of the diameters; MRD, minimal residual disease; IgM, immunoglobulin M.

A significant improvement in BM tumor burden was observed in WM patients as the number of induction courses increased. The median level of tumor burden by MFC was 16.5% (range, 0.3–96.1%) at baseline and was reduced to 0.01% (range, 0%–9.8%) when the best response was achieved. During the course of induction therapy, the clinical characteristics were also significantly improved as the tumor burden in the BM decreased. The patient experienced rapid and sustained reductions in serum IgM levels. The median IgM level was decreased from 34.1 g/L (range, 6.1–122.0 g/L) to 10.0 g/L (range, 0.1–92.1 g/L) after induction therapy. According to the CT scan results of patients with lymphadenopathy, the average sum of the product of the diameters (SPD) at baseline decreased from 8.0 cm^2^ (range, 1.1–38.5 cm^2^) to 1.30 cm^2^ (range, 0–14.8 cm^2^). The median hemoglobin level increased from 84.0 g/L (range, 25–143 g/L) to 136.5 g/L (range, 34–193 g/L) upon the achievement of best response. The platelet counts increased from 141 ×10^9^/L (range, 4–481×10^9^/L) to 180 ×10^9^/L (range, 10–524×10^9^/L) as the number of induction courses increased (**Figure 1**).

### 3.7 Improvement in clinical response in groups with different MRD status

**Figure 2:**
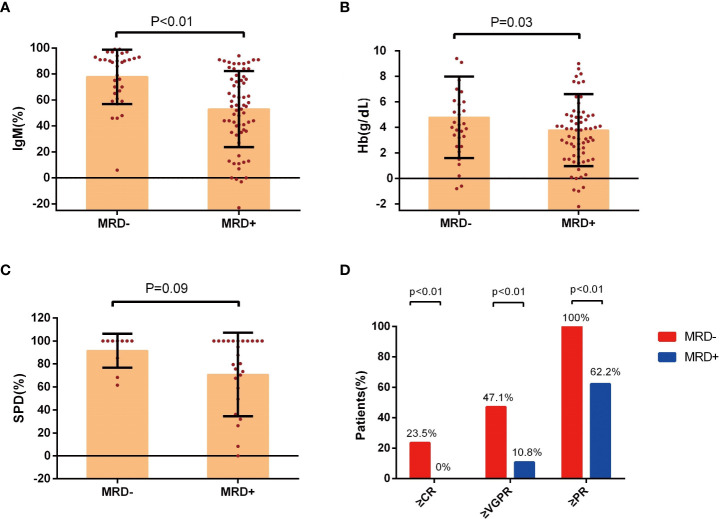
Improvement in clinical response in groups with different MRD status. **(A)** Percentage change of serum IgM levels, **(B)** change of hemoglobin levels, and **(C)** the percentage change of SPD of lymphadenopathy at the time of best response in the patients with Waldenström’s macroglobulinemia were stratified according to the MRD status. **(D)** The response assessment at the time of best response according to the MRD status. IgM, immunoglobulin M; Hb, hemoglobin; SPD, the sum of the product of the diameters; MRD, minimal residual disease; CR, complete response; VGPR, very good partial response; PR, partial response.

IgM reduction (*P*<0.01, **Figure 2A**) and serum hemoglobin recovery (*P*=0.03, **Figure 2B**) were associated with MRD status. Improvements were more evident in patients with uMRD compared with that in MRD-positive patients. The change in the SPD of lymphadenopathy was also greater in uMRD patients compared with that in MRD-positive patients (*P*=0.09, **Figure 2C**). The above results showed the significant consistency between clinical and BM responses.

The rate of PR or better in the uMRD group was 100%, which was significantly higher than that in the MRD-positive group (*P*<0.01). The rates of VGPR or better were 47.1% and 10.8% in the uMRD group and MRD-positive group, respectively (*P*<0.01). The uMRD patients also had a higher CR rate compared with that of the MRD-positive patients (23.5% vs. 0%, *P*<0.01, **Figure 2D**).

### 3.8 Concordance between MRD and BM biopsy

After achieving the best response, 90 of 108 patients underwent a concurrent assessment of MRD status by MFC and BM biopsy samples by immunohistochemistry. The absence of tumor cells was detected in 32% and 41% of MRD samples as shown on MFC and BM biopsy, respectively. A moderate concordance rate of 79% was calculated between MRD status by MFC and BM biopsy status (Cohen’s κ coefficient: 0.55, **Figure S3**), despite 19 cases (21%) showing discordant results (14 patients with positive MRD by MFC but negative MRD by BM biopsy and five patients with negative MRD by MFC but positive MRD by BM biopsy).

### 3.9 MRD status as a predictor of survival

The median follow-up time was 30.8 months (range, 3.5–143 months). The estimated median PFS was 51 months (95% confidence interval (CI): 34.4–67.6 months). The median OS was not achieved.

**Figure 3:**
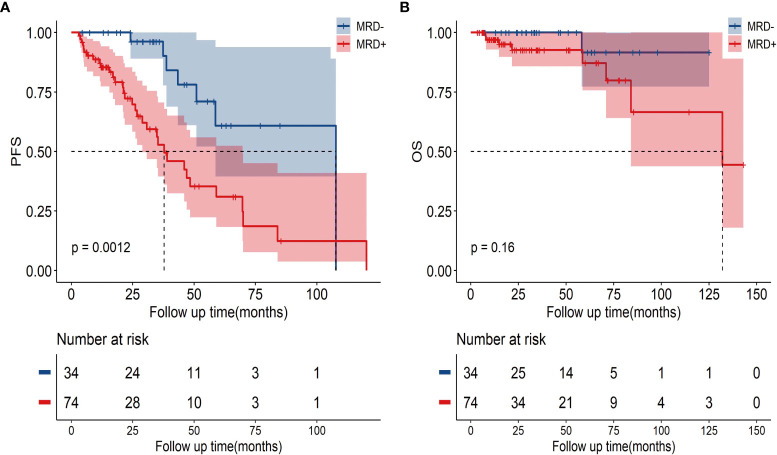
The PFS and OS in uMRD patients and MRD-positive patients in the bone marrow. **(A)** PFS according to MRD status in all patients. **(B)** OS according to MRD status in all patients. PFS, progression-free survival; OS, overall survival; MRD, minimal residual disease.

The uMRD patients had a superior PFS compared with the MRD-positive patients (3-year PFS, 96.2%, [95% CI: 88.8%–100%] vs. 52.8%, [95% CI: 37.5%–68.1%]; *P*=0.0012, **Figure 3A**). However, no significant difference was observed in the OS; the 3-year OS rates were 92.6% (95% CI: 85.5%–99.7%) in the MRD-positive group and 100% (95% CI: 100%–100%) in the uMRD group (P=0.16; **Figure 3B**).

**Figure 4:**
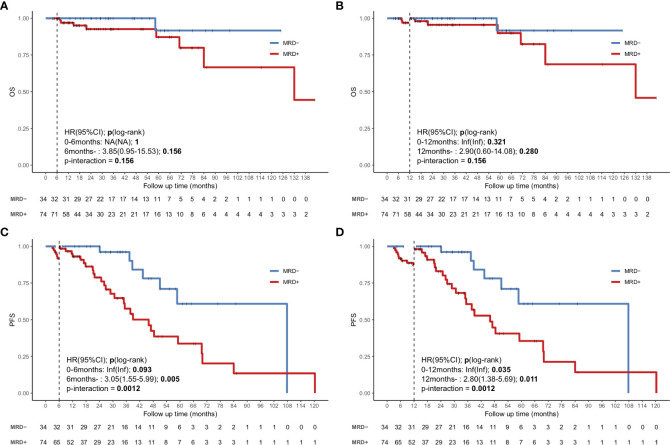
Landmark analysis discriminating PFS and OS in uMRD patients and MRD-positive patients in the bone marrow. **(A, B)** Landmark analysis of OS between events occurring before and after 6 months of follow-up. **(C, D)** Landmark analysis of PFS between events occurring before and after 12 months of follow-up. HR, Hazard ratio.

To avoid immortal time bias, we evaluated the survival using a landmark analysis at 6 and 12 months from the initiation of induction therapy. The landmark analysis of OS in the uMRD and MRD-positive groups also showed no significant difference at 6 and 12 months (**Figures 4A, B**). However, the landmark analysis of PFS in patients with different MRD statuses showed that the uMRD patients had slightly better PFS than the MRD-positive patients (*P*=0.093) within the first 6 months and in the subsequent months (hazard ratio (HR): 3.05, 95% CI: 1.55–5.99, *P*=0.005). The landmark analysis of PFS at 12 months showed a similar result (at 12 months: *P*=0.035; after 12 months = HR: 2.8, 95% CI: 1.38–5.69, *P*=0.011) (**Figures 4C, D**).

### 3.10 Univariate and multivariate analyses

In the univariate analyses, the factors associated with an inferior PFS included aged >65 years (HR: 2.11, 95% CI: 1.30–6.06, *P*=0.01), hemoglobin level of ≤11.5 g/L (HR: 7.08, 95% CI: 1.20–6.77, *P*=0.02), and MRD-positive status (HR: 3.46, 95% CI: 1.54–5.38, *P*<0.01). In the multivariate analyses, only the MRD-positive status (HR: 2.55, 95% CI: 1.10–5.91, *P*=0.03) was an independent adverse factor of PFS (**Table 2**). We also included other prognostic factors in the multivariate analyses, such as IPSSWM, and found that MRD remained an independent poor prognostic factor (**Tables S1, S2**).

**Table 2:** Univariate and multivariate analyses of prognostic factors for PFS.

Variable	Parameter	Univariate analysis		Multivariate analysis	
		HR (95% CI)	*P*	HR (95% CI)	*P*
Age	≤65	1	**0.01**	1	0.06
	>65	2.11 (1.30-6.06)		1.99 (0.97-4.08)	
Gender	Female	1	0.90		
	Male	1.04 (0.54-2.04)			
ECOG	0-1	1	0.62		
	≥2	1.34 (0.37-5.34)			
B symptoms	Absent	1	0.76		
	Present	1.15 (0.50-2.65)			
Hb	>11.5g/dL	1	**0.02**	6.31 (0.84-47.17)	0.07
	≤11.5g/dL	7.08 (1.20-6.77)			
PLT	>100×10^9^/L	1	0.45		
	≤100×10^9^/L	1.33 (0.65-2.68)			
β2-MG	≤3mg/L	1	0.09		
	>3mg/L	2.01(0.92-3.90)			
LDH	<250U/L	1	0.43		
	≥250U/L	1.42 (0.56-4.09)			
ALB	≥35g/L	1	0.25		
	<35g/L	1.45 (0.78-2.78)			
IgM	<70g/L	1	0.22		
	≥70g/L	1.73 (0.77-3.71)			
MRD status	Negative	1	**<0.01**	1	**0.03**
	Positive	3.46 (1.54-5.38)		2.55 (1.10-5.91)	

ECOG, Eastern Cooperative Oncology Group; Hb, hemoglobin; PLT, platelet; β2-MG, β2-microglobulin; LDH, lactic dehydrogenase; ALB, albumin; IPSSWM, International Scoring System for Waldenström’s Macroglobulinemia; MRD, minimal residual disease. The bold values indicate that p < 0.05 and the corresponding factors are significantly associated with survival.

Next, we investigated whether MRD positivity had a prognostic value for PFS in different subgroups. A subgroup analysis of PFS showed that the impact of MRD positivity on outcomes was generally consistent with the overall study results (**Figure S4**).

### 3.11 Combined MRD and response assessment

The median PFS in patients who achieved ≥VGPR was 70.0 months (95% CI: 41.0–99.0 months), which was longer than that in patients who achieved PR (51.0 months, 95% CI 33.3–68.7 months), but no significant difference was observed (*P*=0.85). The patients who attained MR or SD had a considerably inferior outcome with a median PFS of 24.8 months (95% CI: 10.4–39.2 months) compared with those who achieved PR (*P*<0.01, **Figure S5A**).

**Figure 5:**
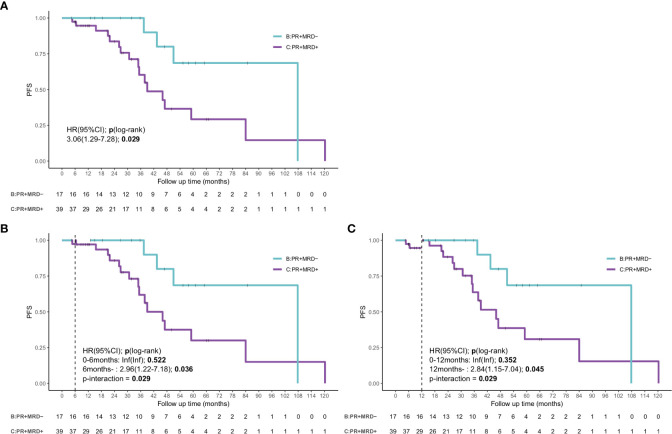
The survival curve and landmark analysis discriminating PFS and OS in uMRD-PR patients and MRD-positive PR patients in the bone marrow. **(A)** The PFS of PR patients according to the status of MRD. **(B)** Landmark analysis of PFS between events occurring before and after 6 months of follow-up. **(C)** Landmark analysis of PFS between events occurring before and after 12 months of follow-up. HR, Hazard ratio.

As most patients achieved PR (51.8%), it remained unclear whether a prognostic difference existed in patients who attained PR. Hence, we used the combination of MRD status and PR for determining the patients’ prognosis, as most patients with ≥VGPR had uMRD. The combination of response assessment and MRD could lead to remarkable differences in the outcomes. The patients with uMRD PR showed a significantly superior outcome, which was comparable to that in patients who achieved ≥VGPR (median PFS: 107.8 months, [95% CI: 51.0–not reached months] vs. 70.0 months, [95% CI: 41.0–99.0 months]; *P*=0.22). The median PFS of the patients with MRD-positive PR was 46.0 months (95% CI 31.3–60.7 months), which was shorter than that of patients with uMRD PR (*P*=0.029, HR: 3.06, 95% CI: 1.29–7.28) (**Figure 5A**). The landmark analyses at 6 and 12 months also showed that the uMRD PR group also had better PFS compared with that of the MRD-positive PR group after 6 months (HR: 2.96, 95% CI: 1.22–7.18, *P*=0.036) or 12 months (HR: 2.84, 95%CI: 1.15–7.04, *P*=0.045) (**Figures 5B, C**). In addition, we analyzed the outcome of MRD-positive PR and MR or SD and found that the median PFS of patients with MRD-positive PR was longer than that of patients with MR or SD, although no significant difference was found (*P*=0.11, **Figure S5B**). Moreover, according to the receiver operating characteristic curve, the 3-year AUC of the combination of MRD and modified IWWM 6^th^ response criteria was slightly higher than that of IWWM 6^th^ response criteria alone; this finding illustrates that the combination of MRD and IWWM 6^th^ response criteria can also effectively predict the PFS, especially in patients who achieved PR (3-year AUC: 0.71 vs. 0.67; **Figure S5C**).

## 4 Discussion

The prognostic significance of MRD status in WM has never been investigated. This study is the first to systematically evaluate the prognostic impact of MRD status on WM in a large cohort. Patients with low levels of hemoglobin and serum albumin, elevated levels of β2-MG, and high-risk IPSSWM stage had an increased likelihood of remaining MRD positive. These characteristics are all identified as predictive factors of poor prognosis, directly or indirectly reflecting the tumor burden (16–20); therefore, it was more difficult for patients with a high tumor load to achieve uMRD. As the number of treatment courses increased, the hemoglobin level and platelet count significantly improved, thus improving the MRD status. Moreover, the uMRD patients displayed significantly better serum IgM responses and changes in hemoglobin level and lymphadenopathies compared with the MRD-positive patients. The above findings showed that MRD response could reflect the dynamic changes of tumor burden in BM and the level of serum IgM or hemoglobin in real time.

The IgM levels decreased independently of the changes in BM response to different therapies (21–24). One possible reason for this discordance is the half-life of IgM and the IgM flare caused by some treatments, such as rituximab-based treatment (6, 7). Therefore, the IgM level does not accurately reflect the early response to treatment (25). In addition, delayed reduction of IgM levels may be associated with residual tumor cells (26), while uMRD in our study represented the clearance of tumor cells, which might help explain the accordance between MRD results and reduction in IgM levels. Consistent with the above findings, an improvement in IWWM-6 responses was observed in the uMRD group compared with that in the MRD-positive group. The CR, ≥VGPR, and ≥PR rates were significantly higher in the uMRD group, and all patients who achieved CR (n=8) had uMRD. In the present study, we confirmed that uMRD was significantly associated with longer PFS; this finding is consistent with that of previous studies, which showed that WM patients achieving deep responses have superior PFS (6, 27, 28). To avoid immortal time bias, we used landmarks to analyze the PFS and OS at 6 and 12 months after induction therapy. The landmark cutoff was set according to the median and maximum times of patients who achieved uMRD. After excluding the immortal time bias, the PFS in the uMRD group was significantly better than that in the MRD-positive group.

Most importantly, we found that MRD-positive status was associated with worse PFS in the whole population, and patients with this status achieved PR. In the two subgroups, this unfavorable outcome occurred regardless of sex, B symptoms, β2-MG level, serum albumin level, and treatment used. In the multivariate analyses, MRD-positive status was a powerful independent predictor of PFS. García-Sanz et al. (29) also demonstrated that the persistence of BM tumor cells detected by MFC after therapy is associated with inferior PFS. However, this study only showed the effect of the presence of residual monoclonal B cells on the prognosis of WM in a small number of samples, and only an extremely few of the patients achieved MRD negativity. Therefore, the value of uMRD in WM has not been fully explored. In addition, alternative technologies with high sensitivity are used for MRD assessment, including polymerase chain reaction (PCR). A previous study demonstrated that MYD88 detection by droplet digital PCR (ddPCR) can be utilized for MRD monitoring, which will become a feasible tool for tumor load screening (30). However, studies that performed continuous MRD monitoring are limited, and this study did not analyze the prognostic impact of MRD status. Moreover, because MRD monitoring by ddPCR relies on the identification of the MYD88^L265P^ mutation at diagnosis, it cannot be applied to approximately 10% of the population who do not carry the MYD88^L265p^ mutation (31). Hence, further prospective studies are warranted to determine the value of MRD monitoring using ddPCR. Therefore, novel technologies should be developed and used in combination with existing indicators to predict the survival of patients with WM. In addition, CXCR4 is a poor prognostic factor for WM patients. But unfortunately, due to the limitations of detection technology, the status of CXCR4 was not available in this study. In the future, we will further explore the impact of CXCR4 on MRD.

POD24 is an important factor in predicting the inferior outcomes in patients with lymphomas, such as follicular lymphoma (32), marginal zone lymphoma (33), and peripheral T-cell lymphoma (34). In the present study, POD24 was a powerful prognostic indicator for OS in patients with WM; this finding indicated that early progression was associated with poor outcomes (**Table S3**). MRD positivity was strongly correlated with early disease progression. None of the 34 uMRD patients had POD24, while 21.6% (16/74) of the MRD-positive patients progressed within 24 months (*P*<0.01).

Normal BM biopsy was used to determine the clinical response to CR based on the IWWM-6 response criteria. However, it is difficult to distinguish malignant cells from normal counterpart cells in patients who have a low number of residual malignant cells in the BM after treatment. Flow cytometry can distinguish aberrant cells from normal cells even at low frequencies based on the abnormal expression of cell surface markers, which is a more sensitive and appropriate technique to monitor response. In the present study, the positivity rate of residual BM infiltration using MFC was 68%, which was higher than the 59% positivity rate using BM biopsy. A comparison of the results of malignant cell detection using MFC and BM biopsy showed moderate concordance. Only a small proportion of cases were MRD positive by MFC, but negative by BM biopsy. This result was somewhat inconsistent with the findings of Barakat et al. (26), which reported that the percentage of residual plasma cells is higher when identified by BM biopsy compared with that by MFC. However, they used three- or four-color MFC to assess the number of malignant cells, which is less sensitive compared with the current eight-color MFC technology. We further evaluated the impact of BM biopsy (*P*=0.04) on predicting WM outcomes, which was also associated with PFS (**Figure S6A**). However, the AUC of MRD by MFC was higher than that of MRD by BM biopsy (3-year AUC, 0.68 vs. 0.58, **Figure S6B**), thus suggesting that MRD monitoring using MFC had a better ability to predict the survival compared with MRD monitoring using BM biopsy.

Due to its indolent characteristics, the therapeutic goal is to control this disease rather than cure it (35). Attaining a deeper response is associated with a better outcome, and VGPR is considered a valid treatment endpoint (36–38). However, despite the substantial progress in therapeutic approaches and excellent response rates among WM patients, only 20%–30% of patients can achieve deep remissions (≥VGPR) (27, 39, 40). It remains largely unclear whether a prognostic difference exists among patients who attain PR. In our cohort, patients with uMRD PR had a considerably longer PFS compared with those with MRD-positive PR, which had a comparable outcome to patients with VGPR and CR. The enhanced competence to identify superior outcomes in patients with PR supported the use of MRD monitoring as an efficient measure for determining the prognosis of WM. Moreover, the combination of MRD detection at the achievement of best response and modified IWWM-6 response criteria can be used as an alternative method to assess prognosis, especially in patients who achieved PR. This study was the first to combine sensitive and comprehensive monitoring of tumor cells together with the evaluation of serum IgM levels to determine the prognosis of WM.

In conclusion, our study showed that assessment of MRD status is a sensitive method for evaluating the treatment efficacy and an independent prognostic factor for PFS. However, further evaluations on a prospective series of patients are required to assess whether MRD can be used for response assessment.

## Data availability statement

The original contributions presented in the study are included in the article/**Supplementary Material**. Further inquiries can be directed to the corresponding authors.

## Ethics statement

Written informed consent was obtained from the individual(s) for the publication of any potentially identifiable images or data included in this article.

## Author contributions

WX and SY designed the research. WX, ZW, and TW analyzed data, performed statistical analyses and wrote the manuscript. YiY, YH, HS, JC, RL, HW, YuY, and QW collected data. WL, GA, WS, WH, and DZ acquired data and managed patients. ZX, JW, and GO suggested revisions. LQ and SY revised the manuscript critically, and approved the final version. All authors contributed to the article and approved the submitted version.
