# Supplementary material for: Minimal residual disease status improved the response evaluation in patients with Waldenström’s macroglobulinemia
Source: Front Immunol. 2023 May 10;14:1171539. doi: 10.3389/fimmu.2023.1171539 (PMC10206219; doi:10.3389/fimmu.2023.1171539)
Supplement: Supplementary file 1 [file DataSheet_1.docx]

**Supplementary material**

**Table S1 Univariate and multivariate analyses of prognostic factors for PFS**

| Variable | Parameter | Univariate analysis | | Multivariate analysis | |
| --- | --- | --- | --- | --- | --- |
|  |  | HR (95 % CI) | *P* | HR (95 % CI) | *P* |
| Age | ≤65 | 1 | **0.01** | 1 | **0.01** |
|  | >65 | 2.11 (1.30-6.06) |  | 3.18 (1.29-7.82) |  |
| Gender | Female | 1 | 0.90 |  |  |
|  | Male | 1.04 (0.54-2.04) |  |  |  |
| ECOG | 0-1 | 1 | 0.62 |  |  |
|  | ≥2 | 1.34 (0.37-5.34) |  |  |  |
| B symptoms | Absent | 1 | 0.76 |  |  |
|  | Present | 1.15 (0.50-2.65) |  |  |  |
| Hb | >11.5g/dL | 1 | **0.02** | 6.75 (0.55-82.58) | 0.14 |
|  | ≤11.5g/dL | 7.08 (1.20-6.77) |  |  |  |
| PLT | >100×10^9^/L | 1 | 0.45 |  |  |
|  | ≤100×10^9^/L | 1.33 (0.65-2.68) |  |  |  |
| β2-MG | ≤3mg/L | 1 | 0.09 |  |  |
|  | >3mg/L | 2.01(0.92-3.90) |  |  |  |
| LDH | <250U/L | 1 | 0.43 |  |  |
|  | ≥250U/L | 1.42 (0.56-4.09) |  |  |  |
| ALB | ≥35g/L | 1 | 0.25 |  |  |
|  | <35g/L | 1.45 (0.78-2.78) |  |  |  |
| IgM | <70g/L | 1 | 0.22 |  |  |
|  | ≥70g/L | 1.73 (0.77-3.71) |  |  |  |
| IPSSWM | Low-risk | 1 |  | 1 |  |
|  | Median-risk | 3.76 (1.04-8.51) | **0.05** | 1.37 (0.23-8.32) | 0.73 |
|  | High-risk | 6.15 (1.69-12.07) | **<0.01** | 1.33 (0.21-8.33) | 0.76 |
| MRD status | Negative | 1 | **<0.01** | 1 | **<0.01** |
|  | Positive | 3.46 (1.54-5.38) |  | 4.25 (1.43-12.64) |  |

ECOG, Eastern Cooperative Oncology Group; Hb, hemoglobin; PLT, platelet; β2-MG, β2-microglobulin; LDH, lactic dehydrogenase; ALB, albumin; IPSSWM, International Prognostic Scoring System for Waldenström’s Macroglobulinemia; MRD, minimal residual disease. The bold values indicate that p < 0.05 and the corresponding factors are significantly associated with survival.

Table S2 Univariate and multivariate analyses of prognostic factors for PFS

| Variable | Parameter | Univariate analysis | | Multivariate analysis | |
| --- | --- | --- | --- | --- | --- |
|  |  | HR (95 % CI) | *P* | HR (95 % CI) | *P* |
| Gender | Female | 1 | 0.90 |  |  |
|  | Male | 1.04 (0.54-2.04) |  |  |  |
| ECOG | 0-1 | 1 | 0.62 |  |  |
|  | ≥2 | 1.34 (0.37-5.34) |  |  |  |
| B symptoms | Absent | 1 | 0.76 |  |  |
|  | Present | 1.15 (0.50-2.65) |  |  |  |
| LDH | <250U/L | 1 | 0.43 |  |  |
|  | ≥250U/L | 1.42 (0.56-4.09) |  |  |  |
| ALB | ≥35g/L | 1 | 0.25 |  |  |
|  | <35g/L | 1.45 (0.78-2.78) |  |  |  |
| ISSWM | Low-risk | 1 |  | 1 |  |
|  | Median-risk | 3.76 (1.04-8.51) | **0.05** | 2.99 (0.65-13.82) | 0.16 |
|  | High-risk | 6.15 (1.69-12.07) | **<0.01** | 3.64 (0.79-16.75) | 0.10 |
| MRD status | Negative | 1 | **<0.01** | 1 | **<0.01** |
|  | Positive | 3.46 (1.54-5.38) |  | 4.59 (1.54-13.73) |  |

ECOG, Eastern Cooperative Oncology Group; LDH, lactic dehydrogenase; ALB, albumin; IPSSWM, International Prognostic Scoring System for Waldenström’s Macroglobulinemia; MRD, minimal residual disease. The bold values indicate that p < 0.05 and the corresponding factors are significantly associated with survival.

**Table S3 Univariate and multivariate analyses of prognostic factors for OS**

| Variable | Parameter | Univariate analysis | |  | Multivariate analysis | |
| --- | --- | --- | --- | --- | --- | --- |
|  |  | HR (95 % CI) | *P* |  | HR (95 % CI) | *P* |
| Age | ≤65 | 1 | **<0.01** |  | 1 | **0.02** |
|  | >65 | 10.05 (4.73-93.64) |  |  | 7.16 (1.30-39.46) |  |
| Gender | Female | 1 | 0.61 |  |  |  |
|  | Male | 1.49 (0.35-6.12) |  |  |  |  |
| ECOG | 0-1 | 1 | 0.45 |  |  |  |
|  | ≥2 | 2.96 (0.18-48.59) |  |  |  |  |
| B symptoms | Absent | 1 | 0.59 |  |  |  |
|  | Present | 1.54 (0.27-10.23) |  |  |  |  |
| HBG | >115g/L | 1 | 0.88 |  |  |  |
|  | ≤115g/L | 1.18 (0.17-8.26） |  |  |  |  |
| PLT | >100×10^9^/L | 1 | 0.87 |  |  |  |
|  | ≤100×10^9^/L | 1.14(0.24-5.37) |  |  |  |  |
| β2-MG | ≤3mg/L | 1 | 0.86 |  |  |  |
|  | >3mg/L | 1.15(0.25-5.44) |  |  |  |  |
| LDH | <250U/L | 1 | 0.91 |  |  |  |
|  | ≥250U/L | 1.13 (0.15-8.21) |  |  |  |  |
| ALB | ≥35g/L | 1 | 0.80 |  |  |  |
|  | <35g/L | 1.18 (0.32-4.39) |  |  |  |  |
| IgM | <70g/L | 1 | 0.16 |  |  |  |
|  | ≥70g/L | 3.55 (0.61-20.82) |  |  |  |  |
| IPSSWM | Low-risk | 1 |  |  |  |  |
|  | Median-risk | 1.04 (0.10-11.32) | 0.97 |  |  |  |
|  | High-risk | 2.80(0.42-14.72) | 0.33 |  |  |  |
| Best response MRD | Negative | 1 | 0.16 |  |  |  |
|  | Positive | 3.85 (0.70-11.48) |  |  |  |  |
| POD24 | Negative | 1 | **<0.01** |  | 1 | **0.04** |
|  | Positive | 15.88 (2.81-89.67) |  |  | 6.30 (1.04-38.16) |  |

OS, overall survival; ECOG, Eastern Cooperative Oncology Group; HGB, hemoglobin; PLT, platelet; β2-MG, β2 microglobulin; LDH, lactic dehydrogenase; ALB, albumin; IPSSWM, International Prognostic Scoring System for Waldenström’s Macroglobulinemia; MRD, minimal residual disease. POD24, progression of disease within 24 months. The bold values indicate that p < 0.05 and the corresponding factors are significantly associated with survival.

**
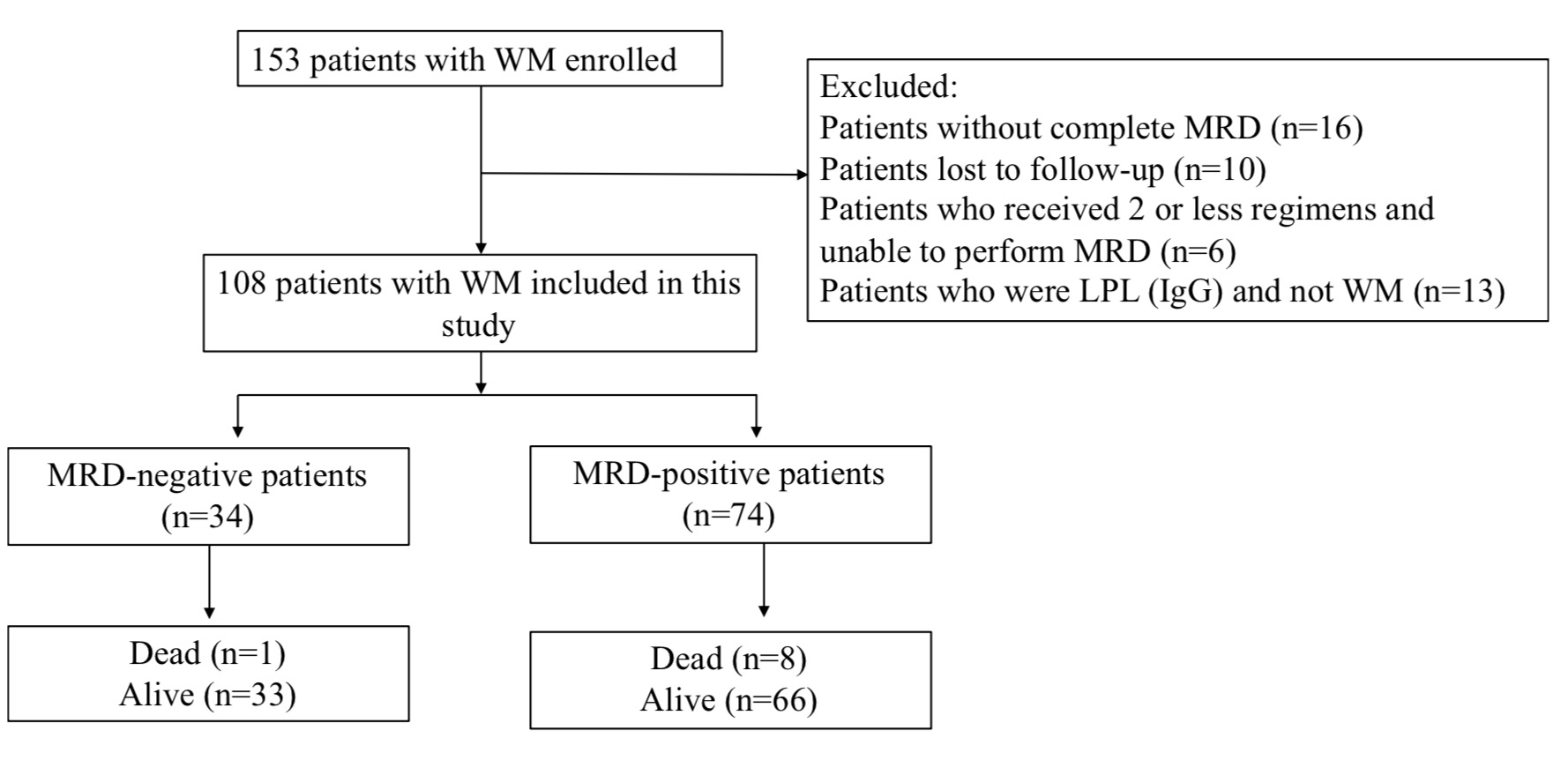
**

**Figure S1 Patient flow chart.**

WM, Waldenstrom’s macroglobulinemia; MRD, minimal residual disease;


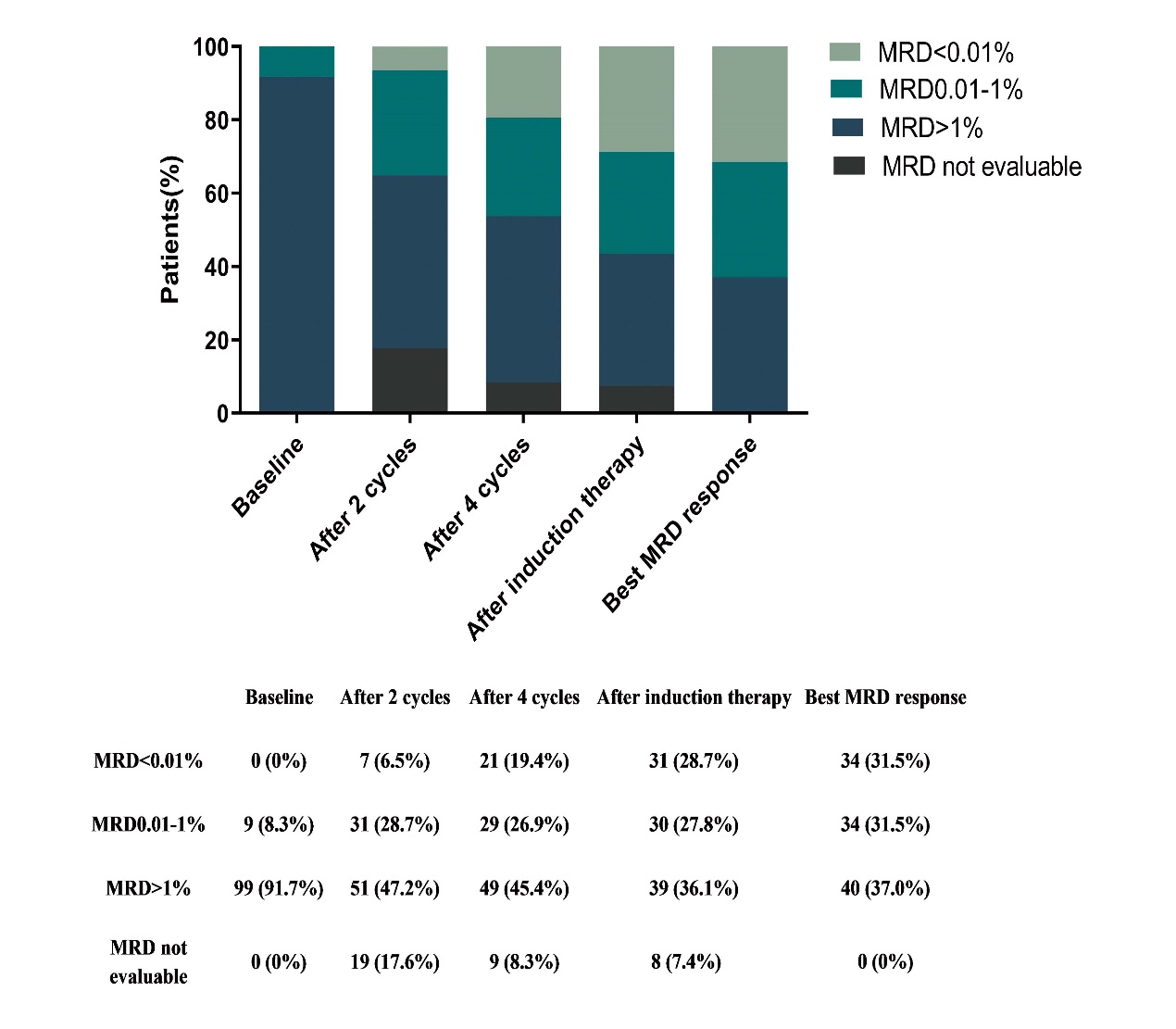


**Figure S2.** **Different minimal residual disease (MRD) levels along with the courses of treatment.**

MRD levels in bone marrow (BM) monitored in 108 patients were deepened with continuous induction treatment.

**
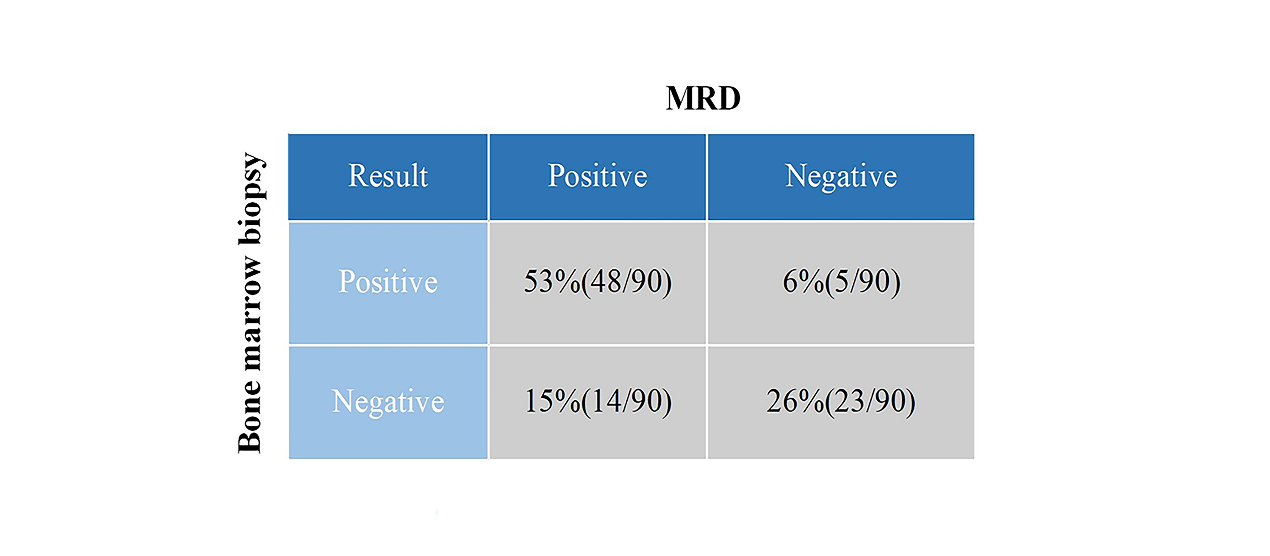
**

**Figure S3 The concordance between MRD and bone marrow biopsy.**

The concordance of residual malignant cells detection between bone morrow biopsy samples using immunohistochemical methods and marrow aspirate samples using flow cytometry. MRD, minimal residual disease by flow cytometry.


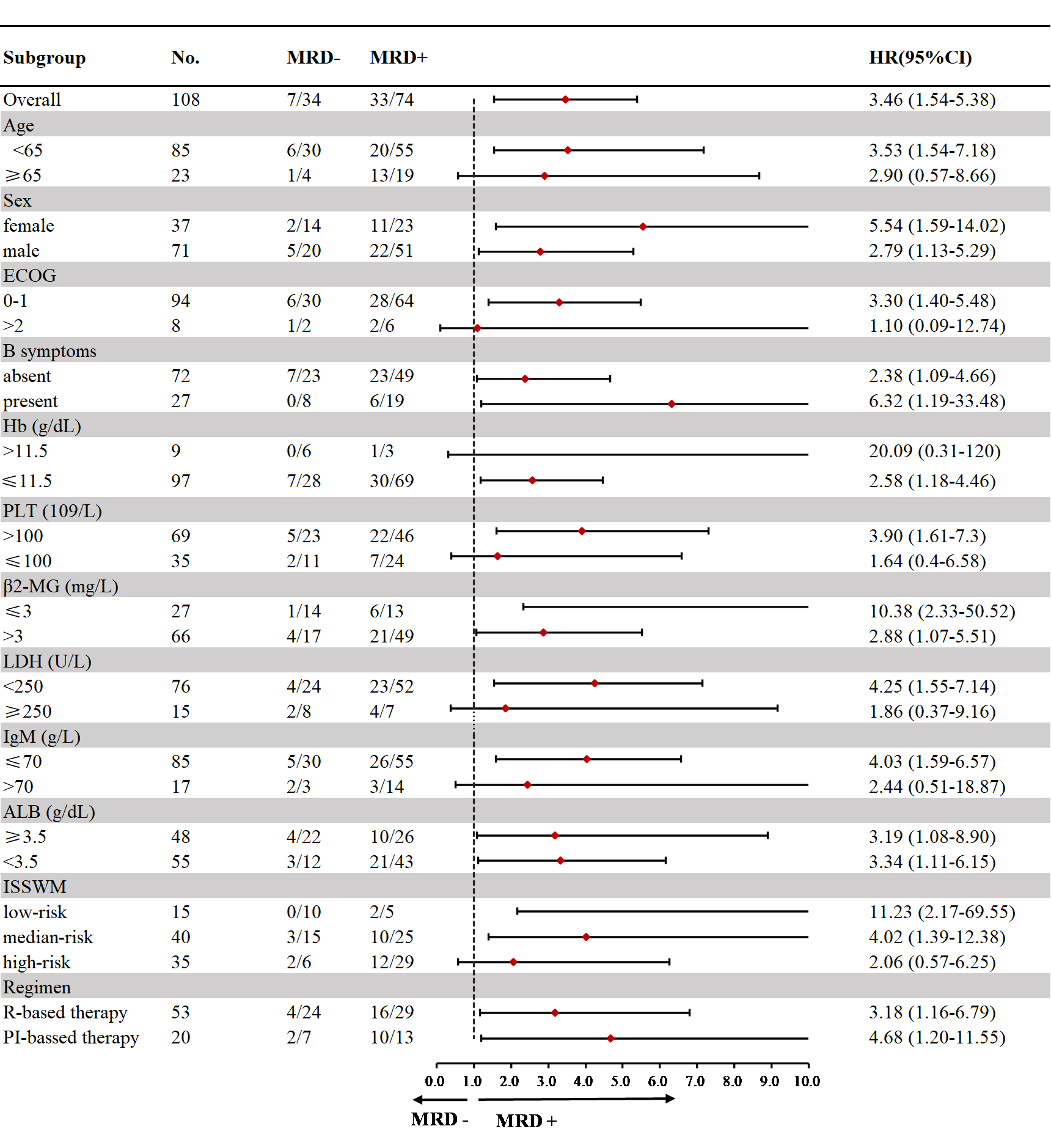


**Figure S4. Forest plots of HRs for PFS according to the MRD status in patients.**

PFS according to the MRD status when other prognostic markers were taken into account. ECOG, Eastern Cooperative Oncology Group; Hb, hemoglobin; PLT, platelet; β2-MG, β2-microglobulin; LDH, lactic dehydrogenase; IgM, immunoglobulin M; ALB, albumin; ISSWM, International Scoring System for Waldenström’s Macroglobulinemia; R, rituximab; PI, proteasome inhibitor; MRD, minimal residual disease.


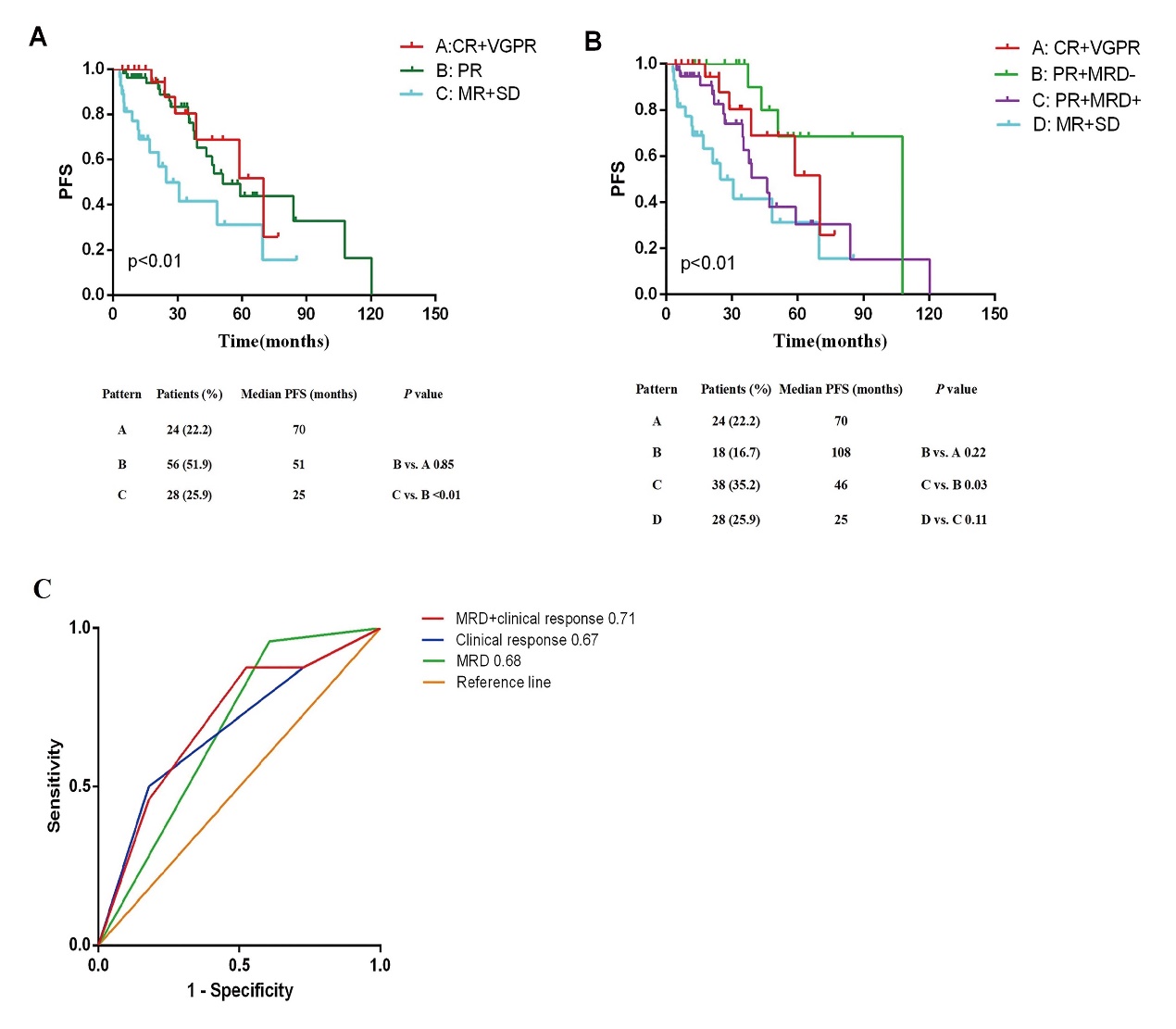


**Figure S5.** **Survival curve of patients with different response assessments.**

(A) PFS of patients according to IWWM-6 response criteria. (B) PFS of patients according to the combination of IWWM-6 response criteria and MRD. (C) Receiver Operating Characteristic (ROC) curves analysis for 3-year PFS for different response assessments. PFS, progression-free survival; IWWM-6, the 6th International Workshop on WM; MRD, minimal residual disease; CR, complete response; VGPR, very good partial response; PR, partial response; MR, minor response; SD, stable disease.

**
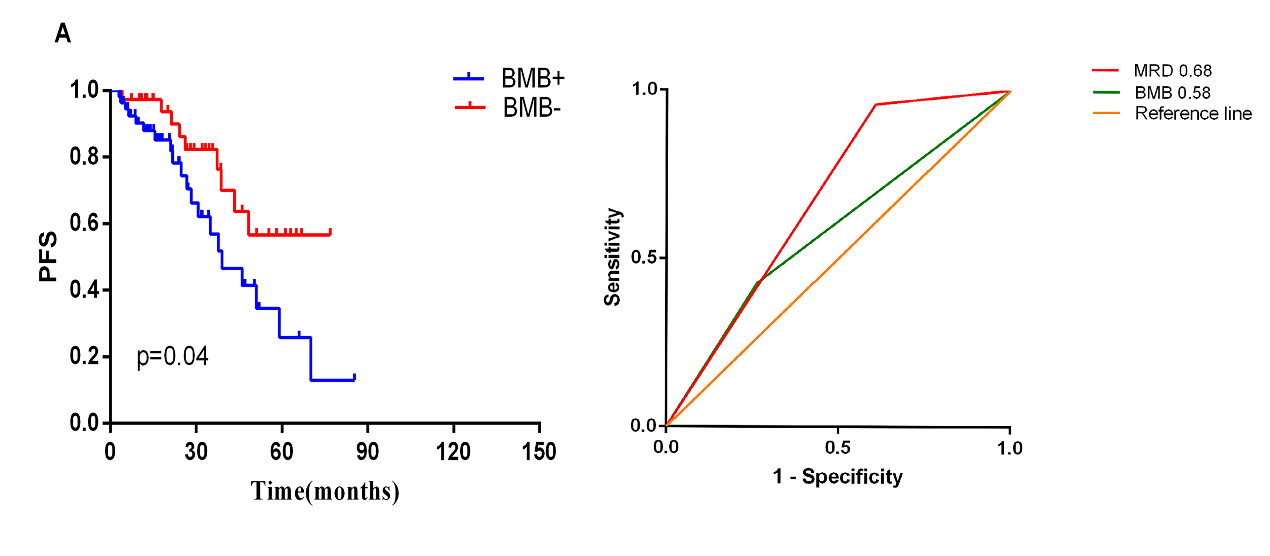
**

**Figure S6** **The prognostic impact of residual malignant cells by bone marrow biopsy on PFS.**

(A)The PFS in residual bone marrow infiltration in bone marrow biopsy samples. (B) ROC curves analysis for 3-year PFS for different bone morrow sample types. MRD, minimal residual disease by flow cytometry; BMB, bone morrow biopsy samples using immunohistochemical methods;
